# Regional-specific effect of fluoxetine on rapidly dividing progenitors along the dorsoventral axis of the hippocampus

**DOI:** 10.1038/srep35572

**Published:** 2016-10-19

**Authors:** Qi-Gang Zhou, Daehoon Lee, Eun Jeoung Ro, Hoonkyo Suh

**Affiliations:** 1 Department of Stem Cell Biology and Regenerative Medicine, Lerner Research Institute, Cleveland Clinic, Cleveland, OH 44195, USA; 2 Institution of Stem Cells and Neuroregeneration, Department of Pharmacology, Pharmacy College, Nanjing Medical University, Nanjing, P.R. China

## Abstract

Hippocampus-dependent cognitive and emotional function appears to be regionally dissociated along the dorsoventral (DV) axis of the hippocampus. Recent observations that adult hippocampal neurogenesis plays a critical role in both cognition and emotion raised an interesting question whether adult neurogenesis within specific subregions of the hippocampus contributes to these distinct functions. We examined the regional-specific and cell type-specific effects of fluoxetine, which requires adult hippocampal neurogenesis to function as an antidepressant, on the proliferation of hippocampal neural stem cells (NSCs). Fluoxetine specifically increased proliferation of NSCs located in the ventral region of the hippocampus while the mitotic index of NSCs in the dorsal portion of the hippocampus remained unaltered. Moreover, within the ventral hippocampus, type II NSC and neuroblast populations specifically responded to fluoxetine, showing increased proliferation; however, proliferation of type I NSCs was unchanged in response to fluoxetine. Activation or inhibition of serotonin receptor 1A (5-HTR1A) recapitulated or abolished the effect of fluoxetine on proliferation of type II NSCs and neuroblast populations in the ventral hippocampus. Our study showed that the effect of fluoxetine on proliferation is dependent upon the type and the position of the NSCs along the DV axis of the hippocampus.

Neural stem/progenitor cells (NSCs) located in the subgranular layer of the dentate gyrus of the hippocampus continuously produce primary projection neurons called dentate granule cells (DGCs) and these adult-born DGCs incorporate into the preexisting hippocampal neural circuits^1^,^2^,^3^,^4^. This hippocampal neurogenesis in the adult brain provides plasticity that has been shown to play a key role in learning and memory^5^. In addition to the role of adult-born DGCs in cognition, it has become clear that hippocampal neurogenesis is also required for the control of emotional status^6^,^7^. Previous seminal studies showed that fluoxetine, a selective serotonin reuptake inhibitor (SSRI), functions as an antidepressant by acting on hippocampal NSCs and thus enhancing neurogenesis^8^,^9^, while the blockage of neurogenesis abolishes the antidepressant function of fluoxetine^9^.

The distinct roles of hippocampal neurogenesis in cognition and emotion have raised an interesting possibility that adult-born DGCs may be functionally heterogeneous. This view has been supported by recent studies suggesting that the hippocampus is anatomically and functionally dissociated along the dorsoventral (DV) or septotemporal axis^10^,^11^,^12^,^13^. Selective ablation of the hippocampal sub-regions followed by behavioral tests, gene expression profiling, and functional imaging analysis strongly suggested that the dorsal (septal pole) hippocampus is involved in spatial learning, navigation, and memory while the ventral (temporal pole) hippocampus may mediate anxiety-related behaviors^14^,^15^,^16^,^17^. Furthermore, regional-specific blockage of neurogenesis by focal x-irradiation supported the possibility that the role of adult-born DGCs in different hippocampus-dependent functions is determined by the position of NSCs along the DV axis: adult-born DGCs in the dorsal hippocampus are required for acquisition of contextual discrimination whereas adult-born DGCs in the ventral hippocampus are necessary for the anxiolytic function of fluoxetine in “non-depressed” mice^18^.

This regional-specific requirement of adult-born DGCs for fluoxetine-mediated antidepressant function raised the possibility that NSCs may differentially respond to fluoxetine depending upon their location along the DV axis of the dentate gyrus of the hippocampus^19^. In this study, proliferation of NSCs in response to fluoxetine was quantitatively analyzed along the DV axis. Our approach showed that fluoxetine specifically increased proliferation of NSCs located in the ventral portion of the hippocampus, but not in the dorsal hippocampus, revealing a positional effect. Within the ventral portion of the hippocampus, fluoxetine specifically induced proliferation of type II NSCs and neuroblasts while mitotic activity of type I NSCs was unaltered. Moreover, epistatic analysis with pharmacological reagents demonstrated that serotonin receptor 1A (5-HTR1A) is a key downstream molecule that mediates the effect of fluoxetine on proliferation of type II NSCs and neuroblasts specifically in the ventral hippocampus. This positional effect on fluoxetine-induced NSC proliferation may be attributed to the contribution of the ventral hippocampus to emotional control.

## Results

### Regional-specific proliferation and survival of newborn cells in response to fluoxetine along the DV axis

**Figure 1:**
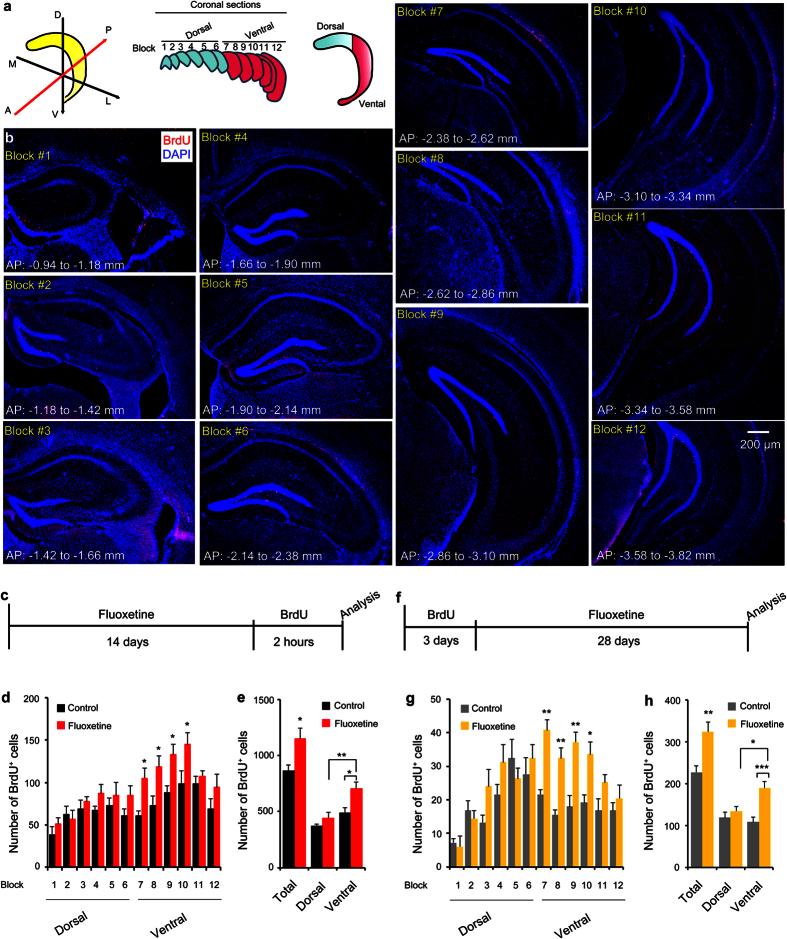
Fluoxetine increases neurogenesis in the ventral part of the hippocampus. (**a**) Three different views of the hippocampus in coronal, sagittal, and horizontal planes (left). A: anterior, P: posterior, D: dorsal, V: ventral, M: medial, L: lateral. Coronal blocks showing anatomical boundaries used for defining sub-regions along the DV axis (middle). The hippocampus was divided into ventral (red) and dorsal (blue) segments (right). (**b**) Representative photos of the DAPI (blue) and BrdU (red) labeled sections in each block along the DV axis. (**c**) The schedule of the BrdU incorporation experiment of proliferation analysis. Bar graph showing the number of BrdU^+^ cells in each block/segment of the hippocampus (**d**) or in the whole hippocampus (**e**) in control and fluoxetine-administered mice. (**f**) The schedule of the BrdU incorporation experiment of survival analysis. Bar graph showing the number of BrdU^+^ cells in each block/segment of the hippocampus (**g**) or in the whole hippocampus (**h**) in control and fluoxetine-administered mice. Data represent the mean ± SEM. *p < 0.05; **p < 0.01; and ***p < 0.001 by two-way ANOVA compared with control mice.

We divided the whole hippocampus into dorsal and ventral segments along the dorsoventral (DV) axis^20^,^21^. The two segments of the hippocampus located at −0.94 to −2.38, and −2.38 to −3.82 millimeters to the bregma were assigned as the dorsal and ventral hippocampus, respectively (Fig. 1a). In this study, we define 6 continuous 40-μm-thick coronal sections as a “block”. Therefore, blocks of 1 to 6, and 7 to 12 represent the dorsal and ventral dentate gyrus of the hippocampus, and every sixth coronal section represents each block (Fig. 1b).

To investigate the regional-specific effect of fluoxetine on proliferation of NSCs along the DV axis of the hippocampus, we treated mice with fluoxetine for 14 days, injected BrdU once, and examined the number of BrdU-expressing cells in each block (Fig. 1c). Consistent with previous results^9^, treatment with fluoxetine increased the number of BrdU-positive cells in the dentate gyrus. Block-by-block analysis of BrdU-positive cells revealed that fluoxetine treatment increased proliferation of NSCs (two-way ANOVA, main effect: fluoxetine, F (1, 84) = 30.64, P < 0.0001), and post-hoc analysis showed that fluoxetine treatment increased the number of BrdU-positive cells specifically in blocks 7 to 10 (Fig. 1d). Thus fluoxetine did not induce proliferation of NSCs uniformly (two-way ANOVA, main effect: fluoxetine treatment, F (1, 14) = 8.524, P = 0.0112), but its effect was specific to NSCs located in the ventral hippocampus (Sidak’s post-hoc analysis, P < 0.05) (Fig. 1e). This selective increase of proliferation in the ventral segment accounts for increased neurogenesis mediated by fluoxetine in the whole hippocampus (t-test, P = 0.0399) (Fig. 1e).

To determine whether fluoxetine treatment enhances the survival of newborn cells in a regional-specific manner, we injected BrdU daily for 3 days, treated the mice with fluoxetine for 28 days, and examined the number of surviving BrdU^+^ cells (Fig. 1f). This long-term treatment with fluoxetine resulted in increased survival of newborn cells. When the number of BrdU^+^ cells was compared in a block-by-block manner, mice treated with fluoxetine for a long-term had a significantly higher number of BrdU^+^ cells (two-way ANOVA, main effect: treatment, F (1, 96) = 43.67, P < 0.0001). Post-hoc analysis showed that the number of BrdU^+^ cells was specifically increased in blocks 7 to 11 while the number of BrdU^+^ cells was not quantitatively different in blocks 1 to 6 (Fig. 1g). A significantly larger number of BrdU^+^ cells were located in the ventral hippocampus of fluoxetine-treated mice compared to control mice (two-way ANOVA, main effect: fluoxetine treatment, F (1, 16) = 21.18, P = 0.0003; Sidak’s post-hoc analysis, the ventral hippocampus, P < 0.0001) (Fig. 1h). Consistent with the previous report^22^, both short-term (14 days) and long-term (28 days) administration of fluoxetine did not alter the area of the dentate gyrus along the DV axis of the hippocampus, indicating that fluoxetine increased the density of dividing NSCs as well as survived cells specifically in the ventral hippocampus (Supplementary Figure 1).

### Fluoxetine specifically acts on actively dividing NSCs in the ventral hippocampus

**Figure 2:**
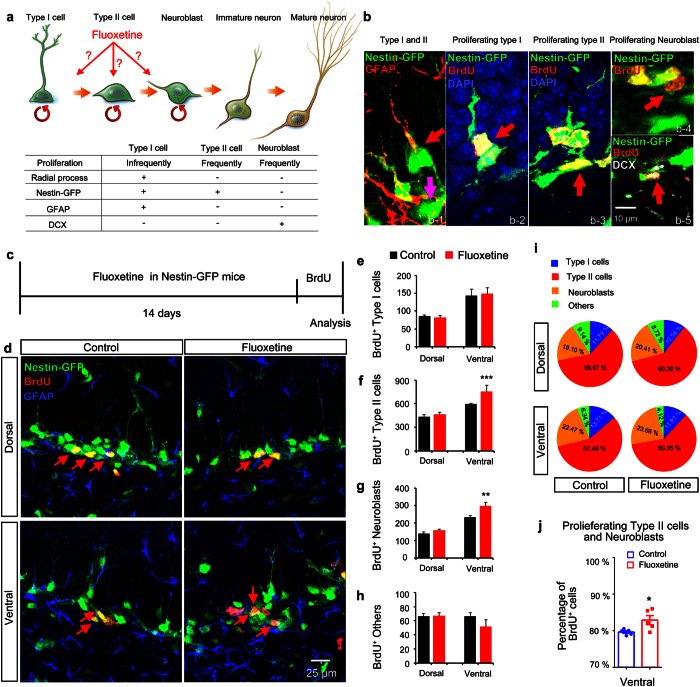
Fluoxetine targets type II NSCs and neuroblasts in the ventral hippocampus. (**a**) Schematic defining different types of NSCs in the neuronal differentiation cascade in the dentate gyrus. (**b**) Representative confocal images of type I cells (b-1 and b-2), type II cells (b-1 and b-3) and neuroblasts (b-4 and b-5). In b1, the red arrow indicates a type I cell with a GFAP-labeled radial process and the purple arrow indicates a type-II cell without a radial process. In b-2 the red arrow indicates dividing type I cells labeled by BrdU antibody. In b-3, the red arrow indicates dividing type II cells labeled by BrdU antibody. In b-4, the red arrow indicates BrdU^+^ neuroblasts not expressing Nestin-GFP. The BrdU^+^ neuroblasts do not have processes and are labeled by DCX in b-5 (red arrow). (**c**) The schedule of the BrdU incorporation experiment. (**d**) Representative confocal images showing the state of type I cells, type II cells and neuroblasts in each segment of the dentate gyrus in mice with or without fluoxetine treatment. Red arrows indicate proliferating type II cells. Bar graph showing the effect of fluoxetine on the number of proliferating type I cells (**e**), type II cells (**f**), neuroblasts (**g**), and others (**h**). (**i**) Pie charts showing the portion of type I cells, type II cells, neuroblasts, and others in each segment along the DV axis of the dentate gyrus in mice with or without fluoxetine treatment. (**j**) Bar graph showing the percentage of BrdU^+^ type II cells and neuroblasts among the BrdU^+^ cells in the ventral segment of control and fluoxetine-administeredrated mice. Data represent the mean ± SEM. *p < 0.05; **p < 0.01; and ***p < 0.001 by two-way ANOVA compared with control mice.

Different types of NSCs defined by their morphology, proliferation kinetics and different marker expression are present in the dentate gyrus of the hippocampus^22^,^23^,^24^,^25^ (Fig. 2a,b). Type I NSCs are slowly dividing cells and have a characteristic radial process that crosses the granular layer and ends as elaborated arbors in the molecular layer. The somas of type I NSCs reside in the neurogenic niche of the subgranular zone (SGZ) of the dentate gyrus. Type II NSCs are actively dividing cells, but they do not have radial processes. While a NSC marker, Nestin, is expressed in both type I and type II NSCs, GFAP is only expressed in the radial processes of type I NSCs (Fig. 2b). Neuroblasts are neuronally committed progenitors that are positive for doublecortin (DCX). These three different types of neural stem/progenitors are in a lineage relationship and NSCs sequentially transit in the order of type I, type II, and neuroblasts before they produce dentate granule cells (DGCs) (Fig. 2a,b).

Using a Nestin-GFP transgenic mouse that expresses GFP under the control of the NSC-specific Nestin promoter in combination with a short-term BrdU pulse and chase method, responsiveness of different types of NSCs to fluoxetine was examined^26^ (Fig. 2c,d). Chronic fluoxetine administration did not change the number of type I progenitors labeled with BrdU along the DV axis of the dentate gyrus of the hippocampus (two-way ANOVA, fluoxetine treatment, F (1, 16) = 0.03279, P = 0.8586) (Fig. 2e). However, fluoxetine significantly increased the number of BrdU-positive type II NSCs and neuroblasts selectively in the ventral hippocampus, but not in the dorsal hippocampus (two-way ANOVA, fluoxetine effect on type II NSCs, F (1, 16) = 20.65, P = 0.0003; Post-hoc analysis, type II NSCs in the ventral hippocampus, P < 0.001; two-way ANOVA, fluoxetine effect on neuroblasts, F (1, 16) = 128.1, P < 0.0001; Post-hoc analysis, neuroblasts in the ventral hippocampus, P < 0.01)^22^ (Fig. 2f,g). Other dividing cell types were not affected by fluoxetine treatment (Fig. 2h). No significant difference in the proportion of each type of NSC among the dividing population was found between control and fluoxetine treatment (Fig. 2I); however, the proportion of actively dividing NSCs including both type II cells and neuroblasts was significantly increased by fluoxetine selectively in the ventral hippocampus (t-test, P < 0.05) (Fig. 2j).

### Activation of 5-HTR1A is sufficient to mimic fluoxetine-mediated regional-specific proliferation of NSCs

**Figure 3:**
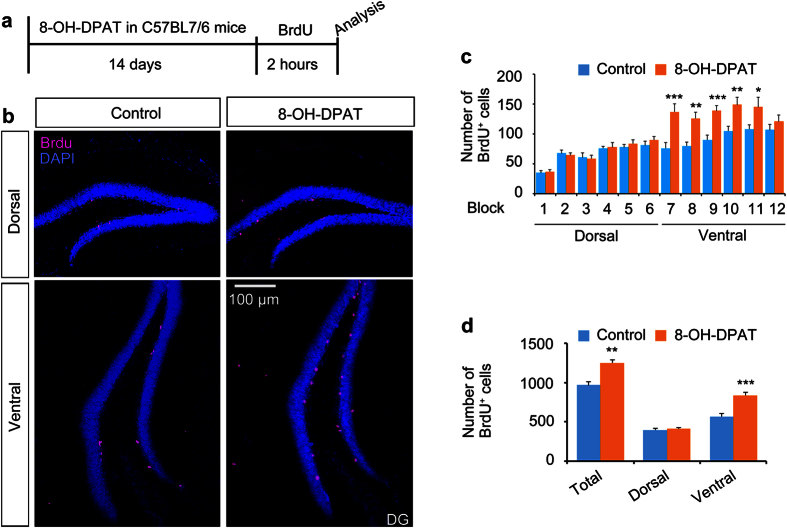
Activation of 5-HTR1A increases neurogenesis in the ventral part of the hippocampus. (**a**) The schedule of the BrdU incorporation experiment. (**b**) Representative confocal images showing BrdU^+^ cells in the dentate gyrus in mice treated with/without 8-OH-DPAT. Bar graph showing the number of BrdU^+^ cells in in each block/segment of the hippocampus (**c**) or in the whole hippocampus (**d**) in control and 8-OH-DPAT-administrated mice. *p < 0.05; **p < 0.01; and ***p < 0.001 by two-way ANOVA compared with control mice.

Next, we investigated whether fluoxetine-induced regional-specific proliferation is mediated by the action of 5-HTR1A. To test this possibility, mice were administered 8-OH-DPAT^27^,^28^, a potent agonist of 5-HTR1A, injected with BrdU once, and then the number of proliferating cells was examined along the DV axis of the hippocampus (Fig. 3a,b). Mice treated with 8-OH-DPAT showed an increased number of BrdU^+^ cells. In a block-by-block analysis, a two-way ANOVA revealed that 8-OH-DPAT differentially affected proliferation of NSCs along the DV axis (F (1, 120) = 20.65, P < 0.0001). Subsequent post-hoc analysis showed that the number of BrdU^+^ cells in block 7 to 11 was significantly higher in 8-OH-DPAT-treated mice while the number of BrdU^+^ cells in block 1 to 6 was comparable between control and 8-OH-DPAT-treated mice (Fig. 3c). The effect of 8-OH-DPAT on the proliferation of NSCs was restricted to the ventral hippocampus while the number of BrdU^+^ cells in the dorsal hippocampus was unchanged (two-way ANOVA, main effect: treatment with 8-OH-DPAT, F (1, 20) = 25.44, P < 0.0001; Sidak’s post-hoc analysis, ventral hippocampus, P < 0.0001), indicating that the action of 5-HTR1A is sufficient to recapitulate the effect of fluoxetine on NSC proliferation in a regional-specific manner (Fig. 3d).

### Action of 5-HTR1A is necessary to mediate the effect of fluoxetine on proliferation of NSCs in a regional-specific and cell-type-dependent manner

**Figure 4:**
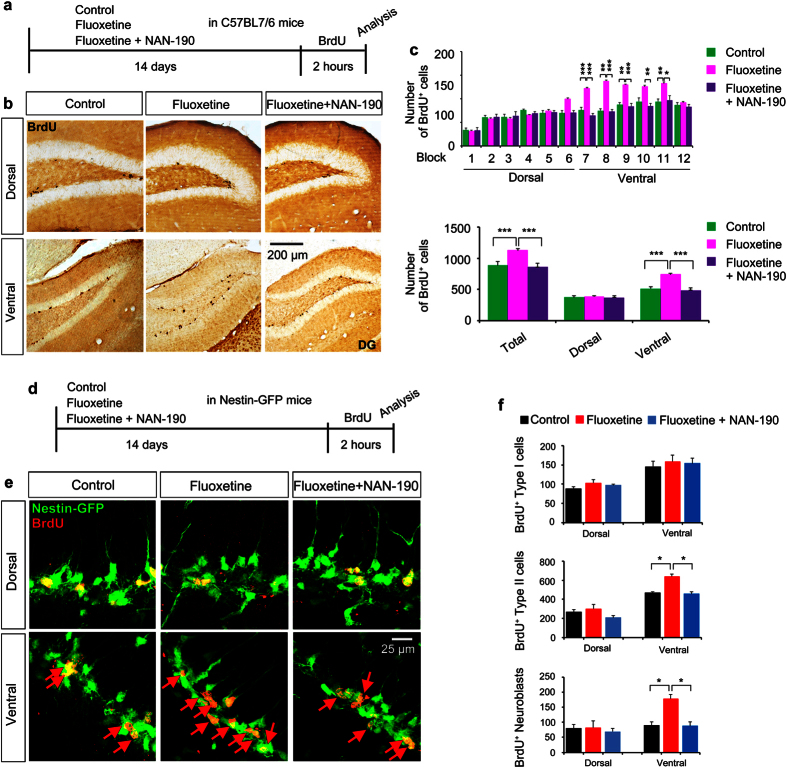
5-HTR1A accounts for the effect of fluoxetine on division of type II cells and neuroblasts in the ventral hippocampus. (**a**) The schedule of the BrdU incorporation experiment of proliferation analysis. (**b**) Representative images showing BrdU^+^ cells in the dentate gyrus in mice treated with fluoxetine alone or fluoxetine and NAN-190. (**c**) Bar graph showing the number of BrdU^+^ cells in the whole hippocampus and in each block/segment of the hippocampus in control and fluoxetine-administrated mice with/without NAN-190 treatment. Data represent the mean ± SEM. (**d**) The schedule of the BrdU incorporation experiment in Nestin-GFP mice. (**e**) Representative confocal images showing the state of type I cells, type II cells and neuroblasts in each segment of the dentate gyrus in control and fluoxetine-administered mice with/without NAN-190 treatment. (**f**) Bar graph showing the effect of fluoxetine on the number of proliferating type I cells, type II cells, and neuroblasts in mice treated with/without NAN-190. *p < 0.05 by two-way ANOVA compared with control mice.

To test whether 5-HTR1A is required to mediate the action of fluoxetine in regional- specific proliferation of NSCs in the ventral hippocampus, we administered NAN-190^27^,^28^, a potent inhibitor of 5-HTR1A, to mice (Fig. 4a). Consistent with our previous results, a block-by-block analysis showed that 14-day-administration of fluoxetine resulted in increased proliferation of NSCs; however, inhibition of the function of 5-HTR1A by NAN-190 effectively normalized fluoxetine-mediated increased proliferation (two way- ANOVA, main effect: treatment, F (3, 180) = 36.84, P < 0.0001; post-hoc analysis, control vs. fluoxetine, P < 0.0001; fluoxetine vs. fluoxetine plus NAN-1=90, P < 0.0001; control vs. fluoxetine plus NAN-190, not significant). Post-hoc analysis revealed that induction of proliferation by fluoxetine and abrogation of this effect by NAN-190 was specific to block 7 to 11 (Fig. 4b,c). This result clearly demonstrated that inhibition of 5-HTR1A by NAN-190 abolished fluoxetine-mediated proliferation of NSCs in the ventral hippocampus (two way- ANOVA, main effect: treatment, F (2, 30) = 34.60, P < 0.0001; post-hoc analysis: in the ventral hippocampus, control vs. fluoxetine, P < 0.0001; fluoxetine vs. fluoxetine plus NAN-1 = 90, P < 0.0001; control vs. fluoxetine plus NAN-190, not significant) (Fig. 4c).

Next, we determined whether NAN-190 abolished the effect of fluoxetine by acting on specific NSC types. Nestin-GFP mice were treated with either fluoxetine or fluoxetine together with NAN-190 for 14 days, and their brains were prepared 2 hours after BrdU injection (Fig. 4d). By using the same criteria used in the previous experiments (Fig. 2), we examined the types of NSCs whose proliferation was affected when they were treated with fluoxetine alone or together with NAN-190. As expected, treatment with fluoxetine specifically increased the number of BrdU^+^ non-radial GFP-expressing cells as well as BrdU^+^ DCX-expressing populations that represent cycling type II NSCs and neuroblasts, respectively, and this fluoxetine-mediated effect was completed abolished by blocking the action of 5-HTR1A with NAN-190 (two-way ANOVA, main effect: treatment on type I NSCs, F (2, 16) = 0.6746, P = 0.5233; main effect: treatment on type II NSCs, F (2, 16), P = 0.0034; main effect: treatment on neuroblasts, F (2, 16) = 5.380, P = 0.0163) (Fig. 4e,f). Post-hoc analysis found that NAN-190 abrogated fluoxetine-mediated proliferation of specific NSC populations in the ventral hippocampus (fluoxetine vs. fluoxetine plus NAN-190 in the ventral hippocampus; type I NSCs, non-significant; type II NSCs, P < 0.05; Neuroblasts, P < 0.05) (Fig. 4f). These results collectively indicate that 5-HTR1A is necessary to mediate the effect of fluoxetine on proliferation of NSCs in a regional-specific and NSC-type-specific manner.

## Discussion

**Figure 5:**
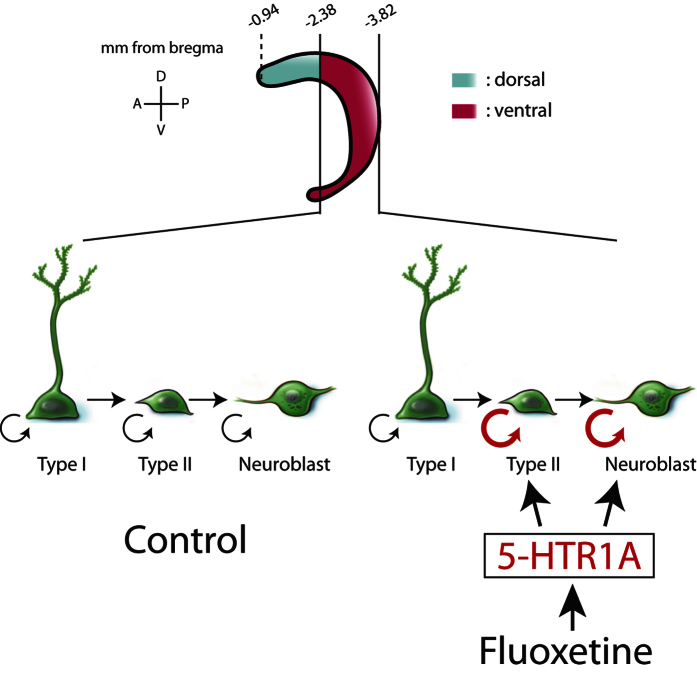
Regional-specific and cell-type-specific effect of fluoxetine on neurogenesis in the ventral hippocampus. Fluoxetine promotes division of type II NSCs and neuroblasts specifically in the ventral hippocampus. 5-HTR1A plays the necessary and sufficient role in mediating the effect of fluoxetine on enhanced neurogenesis in the ventral hippocampus.

It is becoming increasingly apparent that the adult hippocampus is a functionally heterogeneous structure along its DV axis^5^,^11^: The dorsal part of the hippocampus is frequently involved in spatial memory formation and the ventral portion of the hippocampus mediates mood control^29^. Adult-born DGCs produced by neurogenesis have been implicated in both spatial learning and behavioral effects in response to antidepressants such as fluoxetine. This raised an interesting question whether NSCs respond differently to particular extrinsic cues such as fluoxetine depending on their position along the DV axis of the hippocampus^30^. In this study, we addressed this question directly by dividing the hippocampus into two equal segments along the DV axis^19^,^20^,^31^ and investigating the response of NSCs to fluoxetine. Using the property of fluoxetine that induces proliferation of NSCs, thereby increasing the production of hippocampal newborn DGCs, we demonstrated that the position of NSCs with respect to the DV axis of the hippocampus is also an important factor that determines the level of proliferation of NSCs (Fig. 5). Among NSCs, type II and neuroblast populations increased their proliferation in response to fluoxetine and this effect occurred specifically in the ventral hippocampus. Mechanistically, we showed that 5-HTR1A is sufficient and necessary for fluoxetine-mediated induction of NSC proliferation in a regional-specific and NSC cell-type-dependent manner (Fig. 5).

Adult hippocampal neurogenesis is a process that continuously adds new neurons into the hippocampal networks^32^. This persistent production and integration of adult-born DGCs into the system is critical for both cognition and emotion, and disrupted neurogenesis is almost always evident in the pathological conditions associated with cognitive deficits. Hippocampal neurogenesis is a dynamic process that is actively regulated by positive and negative cues^2^. It has been thought that hippocampal NSCs may be a homogeneous population that responds to such signals uniformly. However, our current study strongly suggests that hippocampal NSCs differentially respond to mitotic signals, and thus contribute to distinct functions such as cognition and emotion. For example, both running and fluoxetine induce proliferation of NSCs in the adult hippocampus. However, while running increases proliferation of NSCs mainly in the dorsal hippocampus, as shown in some studies^33^, we show that fluoxetine upregulates NSC proliferation specifically in the ventral hippocampus. Running-induced neurogenesis leads to enhanced performance in spatial learning and fluoxetine-mediated neurogenesis results in relief from the depressed status^9^,^34^. This observation is consistent with the current emerging view that the hippocampus may be functionally dissociated along the dorsal and ventral axis: The dorsal hippocampus is dominantly involved in spatial memory formation and the ventral hippocampus preferentially mediates mood control^10^,^11^. Therefore, it is plausible to conclude that running and fluoxetine specifically target proliferative activity of NSCs located in the dorsal and ventral hippocampus, impacting spatial learning and memory function, and emotional status, respectively. The presence of functionally heterogeneous NSCs along the DV axis may be attributed to the contribution of adult neurogenesis to distinct hippocampus-dependent functions.

Different types of hippocampal NSCs differently respond to fluoxetine in the ventral hippocampus. During neurogenesis, NSCs dynamically transit their status from type I (or QNP: quiescent neural progenitors) to type II (or ANP: amplifying neural progenitors) and from type II to neuroblasts, each of which shows different proliferation kinetics and differential potentials^22^,^24^,^25^. Both type I and type II NSCs are multipotent, but type I NSCs are quiescent compared with the high division rate of type II cells. Type II NSCs produce neuroblasts that are neuronally committed and transiently proliferate to generate DGCs. These cells do not express stem cell markers, but can be defined by double expression of BrdU and DCX. Different factors can regulate proliferation of different populations of NSCs and increased proliferation of any of these NSCs can lead to increased production of neurons. For example, GABA-mediated local neuronal activity specifically targets type I NSCs, keeping their quiescent status, while the loss of GABA transmission to type I NSCs activates their proliferation and symmetric self-renewal^35^. A previous study showed that fluoxetine increased division of type II cells, but the effect of fluoxetine on proliferation of NSCs along the DV axis has not been investigated^22^. Our study showed that fluoxetine specifically increased proliferation of type II cells and neuroblasts, but not type I cells in the ventral part of the hippocampus. This is also consistent with running-induced neurogenesis, which showed an increased proportion of dividing type II and neuroblast cell populations while the overall number of type I and type II NSCs remained unaltered^23^. Thus, while running and fluoxetine target NSCs located in different positions along the DV axis, they appear to target identical types of NSCs.

Previous studies showed that 5-HTR1A mRNA is expressed in the hippocampus, building a dorsal-low to ventral-high concentration gradient^36^ and that 5-HTR1A plays both a necessary and sufficient role in mediating fluoxetine-induced behavior and neurogenic response^9^,^37^. This observation suggested that the action of 5-HTR1A may contribute to a regional-specific and cell-type-dependent response of NSCs to fluoxetine. Indeed, by using pharmacological reagents, we identified 5-HTR1A as a key downstream molecule that mediates the effect of fluoxetine on proliferation of specific NSC types in a regional-specific manner. Administration of a potent activator of 5HTR1A, 8-OH-DPAT, was sufficient to increase proliferation of NSCs. This increase in proliferation is specific to the ventral hippocampus while the mitotic index in the dorsal hippocampus was not affected. Moreover, treatment with a potent inhibitor of 5HTR1A, NAN-190, effectively abolished fluoxetine-mediated proliferation of NSCs in the ventral hippocampus. The action of NAN-190 also normalized the increased cycling type II and neuroblast populations that were induced by fluoxetine, collectively revealing the essential role of 5-HTR1A in mediating fluoxetine-induced proliferation specifically in the ventral hippocampus. Is the function of 5-HTR1A autonomous to NSCs or does 5-HTR1A in other cell types influence the behavior of NSCs? It has been demonstrated that 5-HTR1A is expressed in DGCs; however, the co-expression of 5-HTR1A in NSCs has not been convincingly confirmed. A recent genetic study has provided indirect evidence that the promoter of the 5- HTR1A gene is inactive in NSCs as well as immature newborn DGCs and becomes active when DGCs become mature^38^. Moreover, this study clearly demonstrated that 5-HTR1A in mature DGCs but not NSCs is necessary and sufficient for the behavioral and neurogenic effects of fluoxetine^38^. Thus, the function of 5-HTR1A in the regional-specific and cell-type-dependent response of NSCs to fluoxetine is likely to occur in a non-cell autonomous manner. Although 8-OH-DPAT and NAN-190 have been widely used as an agonist and antagonist for 5-HTR1A^27^,^28^, we should note that the affinity of 8-OH-DPAT and NAN-190 to additional receptors also has been reported^39^,^40^,^41^. A genetic deletion of *5-Htr1a* will be needed to unambiguously confirm the role of 5-HTR1A in the regional-specific and cell-type-specific response of NSCs to fluoxetine.

One important question remains: can our observation that fluoxetine affects proliferation of NSCs in a regional-specific and cell type-dependent manner extrapolate to mice in “depressed” conditions? Recent studies strongly suggest that the effect of fluoxetine on behavior and neurogenic response is dependent upon the state of the animal^18^,^42^. When fluoxetine was applied to two independent animal groups, including “non-depressed” control mice and “depressed” mice, signatures of gene expression in the dentate gyrus between these two experimental groups were distinct^42^. Moreover, addition of a small number of newborn DGCs into the dorsal or ventral hippocampus appears to be sufficient to contribute to cognitive enhancement or fluoxetine-mediated anxiolytic/antidepressant-related behavioral effects in “non-depressed” control mice. However, in a case where animals were situated in a more challenged status such as “depressed conditions”, functional dissociation along the DV axis of the hippocampus appeared to be abrogated and newborn DGCs throughout the entire dentate gyrus were required to mediate fluoxetine-induced behavioral response^18^. These results collectively suggest that the regional-specific and cell-type-dependent effect of fluoxetine on proliferation of NSCs may also be determined by the state of the animal. Future study will be needed to understand the differential response of NSCs to fluoxetine in a state-dependent manner, which will provide an insight to understanding and developing novel antidepressants.

## Methods

### Subjects

All animal procedures were approved by the Institutional Animal Care and Use Committee of the Cleveland Clinic and Nanjing Medical University. All experiments were performed in accordance with relevant guidelines and regulations of the Cleveland Clinic and Nanjing Medical University. Eight - ten weeks old female C57BL/6 mice (purchased from The Jackson Laboratory) were used. Eight - ten weeks old female Nestin-GFP (green fluorescent protein) mice in which the Nestin gene regulatory elements drive the expression of a GFP gene were used in this study^22^. This mouse line has been backcrossed to C57BL7/6 more than 10 generations. Mice were housed in a temperature- and humidity-controlled environment with an alternating 12 hour light and 12 hour dark cycle.

### Treatment with pharmacological reagents

Fluoxetine, (±)−8-Hydroxy-2-(dipropylamino)tetralin hydrobromide (8-OH-DPAT), and 1-(2-Methoxyphenyl)-4-[4-(2-phthalimido)butyl] piperazine hydrobromide (NAN-190) were purchased from Sigma-Aldrich (St Louis, MO, USA) and were dissolved in saline. Fluoxetine (10 mg/kg/d, Sigma-Aldrich, St Louis, MO, USA) was intraperitoneally injected between 10:00 AM-12:00 PM for 14 days. 8-OH-DPAT (0.1 mg/kg/d) and NAN-190 (0.3 mg/kg/d) was intraperitoneally injected. NAN-190 was injected 30 minutes before fluoxetine injection.

### Immunohistochemistry (IHC)

The mice were anesthetized with a mixture of ketamine (100 mg/kg) and xylazine (10 mg/kg) and perfused transcardially with saline followed by 4% paraformaldehyde (PFA). Brains were removed and postfixed overnight in the same solution. To identify the cell types, labeling was carried out on 40-μm free-floating sections as described^43^. Primary antibodies: 5-bromo-2′-deoxyuridine (BrdU) (rat, 1:200; Accurate Chemical & Scientific Corporation, NY), GFP (chicken, 1:100, Aves Labs, OR), glial fibrillary acidic protein (GFAP) (rabbit, 1:200, Dako, CA) were diluted in 0.1 M TBS with 3% normal donkey serum and 0.25% Triton X-100 (TBST) and binding was visualized with a Cy3-conjugated secondary antibody (1:200; Thermo Fisher Scientific Inc, MA). Nuclei were visualized with 4′-6-diamidino-2-phenylindole (DAPI, Sigma-Aldrich, St.Louis, MO). Antigen retrieval steps were used for BrdU staining. Sections were treated with DAPI and fixed in 4% PFA for 10 minutes. Later, sections were treated with 2N HCl at 37 °C, neutralized with 0.1 M boric acid, and blocked. Subsequent steps are identical to those described above (except DAPI treatment). In addition, brain slices also were stained for BrdU with the peroxidase method (ABC system, with biotinylated horse anti-mouse antibodies and diaminobenzedine chromogen; Vector Laboratories). Coronal brain sections (40 μm in thickness) through the entire dentate gyrus were maintained in the serial order. Every sixth section throughout the hippocampus was processed for immunohistochemistry and counting.

### Quantification

Quantification of BrdU^+^ cells in C57BL/6 mice was achieved by counting the number of positively labeled cells in the right side of the dentate gyrus using a 40x objective of an upright microscope (Leica, Germany). One section in each block was counted and reported as a total number of cells in each block by multiplying by 6. Only BrdU^+^ cells in the subgranular zone were counted. For quantification of BrdU^+^ cells in Nestin-GFP mice, fluorescent images of the dentate gyrus were acquired by using a confocal microscope (SP5; Leica, Germany), BrdU^+^ cells located in the subgranular zone were counted, and type I, type II, and neuroblasts were scored based upon the morphology and different expression of markers.

### Measurement of Area

The area of the dentate gyrus in which we quantified cells was measured by using by ImageProPlus5 software (Media Cybernetics, Inc., USA). The area of the dentate gyrus of fluoxetine treated mice was normalized to that of control mice.

### Statistics

Comparisons among multiple groups were performed using two-way ANOVA. Sidak’s multiple comparison method was used for post-hoc analysis. Data are presented as mean ± SEM; *p* < 0.05 was considered statistically significant.

## Additional Information

**How to cite this article**: Zhou, Q.-G. *et al*. Regional-specific effect of fluoxetine on rapidly dividing progenitors along the dorsoventral axis of the hippocampus. *Sci. Rep.* **6**, 35572; doi: 10.1038/srep35572 (2016).

## Supplementary Material

Supplementary Information
