# Supplementary material for: Regional-specific effect of fluoxetine on rapidly dividing progenitors along the dorsoventral axis of the hippocampus
Source: Sci Rep. 2016 Oct 19;6:35572. doi: 10.1038/srep35572 (PMC5069667; doi:10.1038/srep35572)
Supplement: Supplementary Information [file srep35572-s1.pdf]

Regional-specific effect of fluoxetine on rapidly dividing progenitors along the dorsoventral axis of the hippocampus

Qi-Gang Zhou, Daehoon Lee, Eun Jeoung Ro, and Hoonkyo Suh

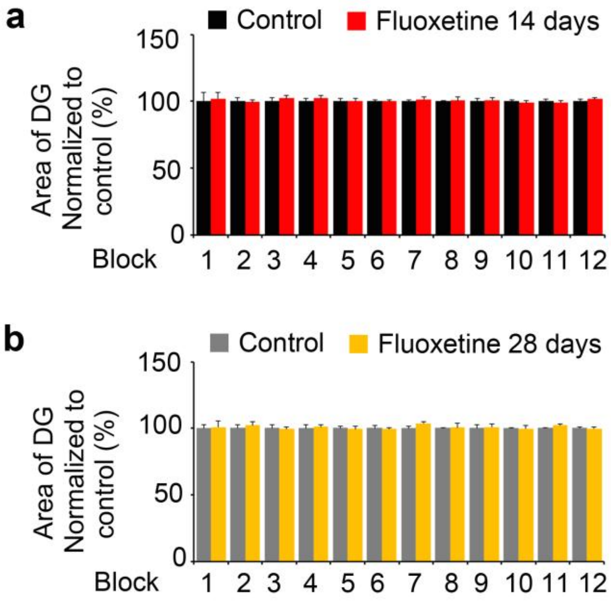

Supplementary Figure 1) Fluoxetine did not change the area of the dentate gyrus along the DV axis. (a) Bar graph showing the area of the dentate gyrus in each block along the hippocampal DV axis of mice treated with or without fluoxetine for 14 days (two-way ANOVA, main effect: fluoxetine treatment, $F(1, 84) = 0.1315$, $P = 0.9996$). **(b)** Bar graph showing the area of the dentate gyrus in each block along the hippocampal DV axis of mice treated with or without fluoxetine for 28 days (two-way ANOVA, main effect: fluoxetine treatment, $F(1, 96) = 0.9344$, $P = 0.3362$). Both short-term and long-term administration of fluoxetine did not change the area of the dentate gyrus of the hippocampus. Data represent the mean ± SEM. Two-way ANOVA compared with control mice.
